# An Integrative Model for the Neural Mechanism of Eye Movement Desensitization and Reprocessing (EMDR)

**DOI:** 10.3389/fnbeh.2016.00052

**Published:** 2016-04-05

**Authors:** Olivier A. Coubard

**Affiliations:** The Neuropsychological Laboratory, CNS-FedParis, France

**Keywords:** anxiety disorders, post-traumatic stress disorder (PTSD), eye movement desensitization and reprocessing (EMDR), attentional control, reaction time

## Abstract

Since the seminal report by Shapiro that bilateral stimulation induces cognitive and emotional changes, 26 years of basic and clinical research have examined the effects of Eye Movement Desensitization and Reprocessing (EMDR) in anxiety disorders, particularly in post-traumatic stress disorder (PTSD). The present article aims at better understanding EMDR neural mechanism. I first review procedural aspects of EMDR protocol and theoretical hypothesis about EMDR effects, and develop the reasons why the scientific community is still divided about EMDR. I then slide from psychology to physiology describing eye movements/emotion interaction from the physiological viewpoint, and introduce theoretical and technical tools used in movement research to re-examine EMDR neural mechanism. Using a recent physiological model for the neuropsychological architecture of motor and cognitive control, the Threshold Interval Modulation with Early Release-Rate of rIse Deviation with Early Release (TIMER-RIDER)—model, I explore how attentional control and bilateral stimulation may participate to EMDR effects. These effects may be obtained by two processes acting in parallel: (i) activity level enhancement of attentional control component; and (ii) bilateral stimulation in any sensorimotor modality, both resulting in lower inhibition enabling dysfunctional information to be processed and anxiety to be reduced. The TIMER-RIDER model offers quantitative predictions about EMDR effects for future research about its underlying physiological mechanisms.

“Remontée en sa chambre, la jeune femme [Jeanne Le Perthuis des Vauds] se demandait comment deux retours aux mêmes lieux qu’elle croyait aimer pouvaient être si différents. Pourquoi se sentait-elle comme meurtrie, pourquoi cette maison, ce pays cher, tout ce qui, jusque-là, faisait frémir son coeur, lui semblaient-ils aujourd’hui si navrants? Mais son œil soudain tomba sur sa pendule. La petite abeille voltigeait toujours de gauche à droite, et de droite à gauche, du même mouvement rapide et continu, au-dessus des fleurs de vermeil. Alors, brusquement, Jeanne fut traversée par un élan d’affection, remuée jusqu’aux larmes devant cette petite mécanique qui semblait si vivante, qui lui chantait l’heure et palpitait comme une poitrine.”

*—(Guy de Maupassant, 1883/2009, see “To Physiology” Section for English version)*.

## Introduction

In 1987, Francine Shapiro observed on herself that rhythmic eye movements may cause cognitive and emotional changes. From that observation, she developed a Cognitive and Behavioral Therapy (CBT)-inspired protocol called Eye Movement Desensitization and Reprocessing (EMDR; initially EMD then EMDR) to treat anxiety disorders, particularly Post-Traumatic Stress Disorder (PTSD; Shapiro, 1989a,b). In this protocol, left-right smooth pursuit eye movements are elicited to alleviate negative cognition, negative emotion, and unpleasant physical sensations associated with a traumatic memory (desensitization phase) and to reinforce positive cognition (reprocessing phase). Specifically, eight phases are distinguished within the procedure: (i) patient history and treatment planning; (ii) preparation; (iii) assessment, which involves defining current negative cognition, future positive cognition and its level of validity on a subjective scale, current negative emotion, related physical sensation(s) and its level of disturbance on another subjective scale; (iv) desensitization; (v) reprocessing, which involves installing positive cognition; (vi) body scanner; (vii) closure; and (viii) reassessment.

After a brief overview of procedural aspects of EMDR and theoretical hypotheses, I will describe how the scientific community is divided about EMDR. Then, I will present theoretical and technical tools currently used in movement research, and explore how such quantitative tools may help to put forth our understanding of EMDR neural mechanism.

## Hypotheses About EMDR Effects

### Procedural Aspects

EMDR is a complex protocol in the sense that it involves several components: behavioral, cognitive, emotional and physical (Shapiro, 2001). For that reason, every component of the protocol is likely to play a role in its benefits. Indeed, several aspects are puzzled in EMDR such as: (i) exposure and desensitization (McNally, 1999); (ii) self-mastery reinforcement (Bandura, 2000); (iii) focus on physical sensations (Gendlin, 1996); (iv) cognitive reprocessing (Meichenbaum and Fitzpatrick, 1993); (v) reconnection of disseminated fragments of traumatic memory and their integration into memory (van der Kolk et al., 2001); (vi) free association as in psychoanalysis (Wachtel, 2002); (vii) full consciousness inspired from meditative practice (Krystal et al., 2002); or (viii) bilateral stimulation (Shapiro, 1994).

First, EMDR effects may be attributed to exposure, which induces desensitization on its own (Lohr et al., 1998; McNally, 1999). However EMDR alternates exposure with debriefing periods. During these periods, the patient is required to communicate on his/her thoughts and feelings (Rennie, 1994; Boudewyns and Hyer, 1996). By alternating exposure with metacognition, EMDR reduces anxiety in shorter time than exposure (Tinker and Wilson, 1999; McCullough, 2002), which cannot be obtained by simply interrupting exposure (Wolpe, 1990).

Second, switching between exposure and metacognition reinforces self-mastery and self-efficacy (Bandura, 2000). Indeed, the patient is forced to face and manipulate his/her negative cognition and emotion thus preventing their avoidance (Shapiro, 2001).

Third, identify physical sensations associated with emotion (e.g., stomach pain associated with fear) and quantify the level of disturbance on a subjective scale—the subjective units of discomfort scale (SUDS; Wolpe, 1973) helps the patient to give sense to his/her emotional state (Gendlin, 1996). It additionally provides both the patient and the practitioner concrete information to assess the level of anxiety throughout the EMDR protocol.

Fourth, cognitive reprocessing is a central aspect of EMDR protocol. The patient is invited to verbalize the negative cognition he/she may covertly express about him/herself, which acts as dysfunctional schemes. As a matter of fact, he/she becomes aware of potential irrational character of such schemes and of their deleterious impact in daily life. During the EMDR protocol, the patient is then invited to replace this negative cognition by a new positive one, which is reinforced using another subjective scale—the validity of cognition (VOC)—to quantify his/her belief in such cognition. This positive cognition acts as new narrative content of self-perception and self-esteem, which facilitates the therapeutic process (Beck, 1967; Meichenbaum and Fitzpatrick, 1993; Young, 1999; Young et al., 2002).

Fifth, one aspect of PTSD is that traumatic memory information is dissociated and disseminated into fragments. Here, we define traumatic memory as a blend of multi-sensory images (visual, auditory, somatosensory, etc.), negative cognition, negative emotion, and unpleasant physical sensations. Those fragments behave as labile and dysfunctional information until they can be reconnected—in other words integrated. The goal of EMDR is specifically to integrate these fragments (van der Kolk, 1994; van der Kolk and Fisler, 1995; Shapiro, 2001). Importantly, the patient may be more or less conscious of such information until it get processed and integrated in consciousness. According to Behavior, Affect, Sensation, Knowledge (BASK) model of dissociation (Braun, 1988), fragment reconnection is done through narrative process and integrated into semantic memory (Braun, 1988; van der Kolk et al., 2001).

Sixth, EMDR is not only compatible with CBT but also with free association of psychoanalysis (Wachtel, 2002), which makes it attractive to any practitioner. During the EMDR protocol, the patient jumps from one memory information to another, which can be separated by years or decades but have in common some dysfunctional node. The main difference between psychoanalysis and EMDR is temporal: what takes years and only accidentally leads to integration in the former, is expediently and efficiently achieved in a few hours in the latter (Rogers and Silver, 2002). This feature may be explained by the ways the two techniques access information: linguistic in psychoanalysis vs. multimodal in EMDR.

Seventh, EMDR is in line with meditative practice (Kabat-Zinn, 1990; Krystal et al., 2002), full attention and full consciousness of dialectic behavioral therapies (Linehan, 1993), and acceptance and commitment therapies (Hayes et al., 1999). According to interaction cognitive systems by Teasdale and Barnard (1993) and Teasdale (1999), the best results are obtained in the intermediate state between over-emotion (mindless emoting) and over-attention (feeless thinking; Greenberg and Safran, 1987; Shapiro, 2001). Such intermediate state is a subtle balance of right-here-and-now attentive feeling of self, leading to harmonious flow of cognition, emotion and physical sensations (Kabat-Zinn, 1990; Servan-Schreiber, 2003).

Eight, a final important property of EMDR protocol is bilateral stimulation. Originally, bilateral stimulation has been obtained through pursuit eye movements in response to the therapist fingers’ movement or using *ad hoc* technical devices to simulate this movement (Shapiro, 2001). Such movements are usually elicited in the horizontal plane (left-right), but they can also use vertical, oblique or ellipsoid trajectories. Importantly, bilateral stimulation cannot only be visuomotor but also auditory (i.e., a sound alternating in left and right ears) or tactile (i.e., a stimulation of any left-right part of the body; Shapiro, 1994, 2001). Therefore bilateral stimulation *per se*, i.e., regardless of sensorimotor modality, seems to be more relevant to EMDR effects than eye movements on their own.

### Theoretical Hypotheses

Besides potential EMDR effects inherent in its procedural aspects, several hypotheses have been advanced to account for the mechanisms underlying EMDR, such as orienting response (Armstrong and Vaughan, 1996), de-arousal (MacCulloch and Feldman, 1996), non-orienting reflex (Wilson et al., 1996), visuospatial sketchpad (Andrade et al., 1997), Rapid Eye Movement (REM)-like movement (Stickgold, 2002).

In orienting response, it has been suggested that, before intervention, access to traumatic memory is only available through a configuration of automatic physiological states (Shapiro, 1991; MacCulloch and Feldman, 1996). EMDR would be able to modify these states by generating imbalance in automatisms and building up opportunities for a reprocessing.

Behavioral models have also suggested that orienting response acts as a learning that breaks with avoidance or flight behaviors (Armstrong and Vaughan, 1996). Cognitive models have emphasized that EMDR may cause an orienting response, which generates imbalance in the traumatic associative network, facilitating information reprocessing associated with progressive positive emotion (e.g., Nathanson, 1996).

In neurobiological models, left-right eye movements would act as REM known to occur during the paradoxical sleep phase and hypothesized to collect episodic recent events and to integrate them into semantic memory (Bergmann, 2000; Stickgold, 2002). Such proposal has received support from experiments showing that left-right eye movements boost episodic but not semantic retrieval (Christman and Garvey, 2000), but it remains unclear how non visuomotor bilateral stimulation modalities (auditory, tactile) would act.

It has been suggested that EMDR may act as hypnosis or some sort of suggestibility. However hypnosis has been rejected to explain EMDR effects for at least three reasons. First EMDR has shown significant results in PTSD, which is not the case of hypnosis (Shapiro, 2001). Second, patients are always vigilant during an EMDR session and they reject suggestions made by the practitioner that are not ecologically acceptable for them, i.e., incompatible with their beliefs, which is not the case of hypnosis (Hekmat et al., 1994). Second, electroencephalographic (EEG) recordings have evidenced differential patterns of oscillations during EMDR (Nicosia, 1995) vs. hypnosis (Sabourin et al., 1990).

The best EMDR effects are produced by the intermediate situation in which the patient is half in the past and half in the present, in other words not too distracted and not too capable of negative association. Distraction is an important feature of attentional control (Parasuraman, 2000). Some authors have suggested that bilateral stimulation distracts the patient from his/her traumatic memory in a way that results in deconditioning. Indeed, distraction would prevent goal-directedness related to PTSD, which consists in negative reinforcement of traumatic memory, and would open the door to reprocessing made of positive cognition and emotion (Dyck, 1993). As a result of this view, Dyck (1993) recommended inducing distraction in the corresponding modality of traumatic memory (e.g., auditory stimulation for auditory components of traumatic memory) and suggested that EMDR may be less efficient for multiple- than for single-event traumas. However, both of these corollaries are not clinically supported (Shapiro, 2001).

A final issue that deserves discussion is the hypothesis according to which EMDR may act as hemispheric synchronization. Under normal conditions, neurological balance is achieved by excitatory and inhibitory mechanisms (Pavlov, 1927), in which positive and negative events are integrated by an adaptive information processing system (AIPS; Shapiro, 2001). Consistent with pioneer (Janet, 1889; Freud, 1919) and neurobiological (van der Kolk, 1994; Stickgold, 2002) proposals, imbalance may be generated in the neurological system when the AIPS is unable to integrate negative events, due to either low capacity of the former or high intensity of the latter or both, thus generating the trauma. The aim of any therapeutic intervention may be to restore the initial neurological balance. In this context, EMDR might stimulate the AIPS by simultaneously activating both hemispheres as a pacemaker thus facilitating down regulation of the limbic system and integration of dysfunctional information in cortical functions (Bergmann, 2000; Shapiro, 2001; Stickgold, 2002).

Such hypothesis is partially based on hemispheric asymmetry in emotional processing. The left hemispatial bias in perceptual face processing is reduced vs. enhanced in high-depressed vs. high-anxious participants, compared to low-depressed and low-anxious participants, respectively (Heller et al., 1995; Keller et al., 2000). EEG has also evidenced a right posterior deficit in depressed patients to positive face stimuli (Deldin et al., 2000). On the other hand, functional brain imaging of PTSD has shown increased activation of the right amygdala and of visual areas consistent with emotional and visual re-experiencing of the trauma, together with decreased activation in the left inferior frontal cortex (Broca’s area), which makes sense with patients’ common report that they find “no words” to tell their story (Rauch et al., 1996; Hull, 2002; Lanius et al., 2004; Shin et al., 2004, 2005).

## The Scientific Community is Divided About EMDR

### Half the Scientific Community is Enthusiastic About EMDR

Since the original report by Shapiro (1989a,b), more than 300 articles and more than 95 review articles have been published about EMDR, splitting the scientific community into enthusiasts and sceptics.

The argument to support EMDR enthusiastically as therapeutic intervention in anxiety disorders can be analyzed according to three levels.

At level 1, qualitative reports and uncontrolled studies (i.e., without control group) have suggested that EMDR might have interesting therapeutic results (e.g., Marquis, 1991; Puk, 1991; Wolpe and Abrams, 1991; Lipke and Botkin, 1992; Oswalt et al., 1993; Pellicer, 1993; Forbes et al., 1994; Spates and Burnette, 1995).

At level 2, other studies have showed that EMDR intervention in the experimental group leads to better outcomes than in the control group without any treatment (e.g., patients on a waiting list). Specifically in PTSD and trauma, several reports have documented that patients benefiting from EMDR as compared to patients without treatment, yield significantly lower SUDS (e.g., Wilson et al., 1995, 1997; Boudewyns and Hyer, 1996; Rothbaum, 1997; Carlson et al., 1998). Among these studies, Rothbaum (1997) additionally showed that EMDR improved the score in another scale, the clinician-administered PTSD Symptom Scale (PSS; Foa et al., 1993), whereas Boudewyns and Hyer (1996) observed EMDR-induced change in heart rate.

Considering other anxiety disorders, EMDR has shown better results than no treatment in spider phobia as evidenced by lower SUDS and higher VOC (Bates et al., 1996; Muris and Merckelbach, 1997), in social phobia (Foley and Spates, 1995) and panic disorder (Feske and Goldstein, 1997) using standardized measures such as the Behavioral Assessment of Speech Anxiety (BASA) and the Beck Depression Inventory (BDI), respectively.

These early studies in EMDR history have had the merit to point out the importance of treatment duration by showing that 5–12 sessions of EMDR are more efficient than only two, as well as the absence of correlation between subjective, psychometric and physiological measures (Cahill et al., 1999). Noticeable treatment speed and effect size have also favored EMDR from that period: 3–5 sessions of EMDR yield effect size of 77%–90% in PTSD/trauma (Rothbaum, 1997; Wilson et al., 1997; Scheck et al., 1998), whereas 7–15 sessions of Stress Inoculation Training with Prolonged Exposure (SIT-PE) associated with more than 110 h of homework yield effect sizes of only 55%–75% (Marks et al., 1998; Foa et al., 1999; Tarrier et al., 1999).

At level 3, EMDR intervention has been found to be superior to non validated treatments. Non validated treatments refer to interventions which are not well defined in their content, and/or not standardized in their methods, and/or not validated by Random Clinical Trials (RCT). Image Habituation Training (IHT; Vaughan and Tarrier, 1992), relaxation, biofeedback are examples of non validated treatments of anxiety disorders. According to several reports, EMDR seems to be more efficient in treating PTSD/trauma than: (i) IHT (Vaughan et al., 1994); (ii) relaxation should it be assisted (Carlson et al., 1998) or not (Silver et al., 1995) by biofeedback techniques; and (iii) active listening of traumatic history (Scheck et al., 1998). More interestingly, EMDR has shown better results in treating PTSD/trauma than some mixture of treatments, which has the quality of being standard and provided by the Health Maintenance Organization (HMO; Marcus et al., 1997).

Taken together, there are arguments in the literature to suggest that EMDR might be a useful strategy for treating PTSD/trauma and eventually other anxiety disorders, when it is compared to a control group without treatment or a control group benefiting from other non validated treatments. However such studies present some limitations in their methodology and the potential impact of their results, which I now discuss.

### Half the Scientific Community is Sceptical About EMDR

In intervention studies, the changes observed between pre- and post-test periods may be attributed to multiple factors: (1) passage of time, which involves healthy aging, natural remission or spontaneous recovery; (2) test-retest effect, which includes learning effect (the fact of repeating the assessment improves performance) and regression to the mean (the tendency of extreme scores in pre-test period to move away from extremes in post-test period); (3) placebo effect, which refers to volition and expectancy (the will and expectation that things will be better improves performance, particularly for subjective measures such as SUDS and VOC), and for which it is worth recalling that it yields on its own to effect size of 30%–40%; (4) trainer effect, which involves dimensions like the quality of relationship between therapist and patient, empathy, healing, and in the extreme the guru effect (a particular therapist showing particular influence that is nevertheless not reproducible with other practitioner); (5) treatment effect without specificity, when the intervention is either complex (like EMDR) or not well defined or when the control group is not specific (i.e., waiting list, no treatment or non validated treatment for targeted disorder); and (6) treatment effect with genuine specificity, when the treatment is compared to previously validated treatment for targeted disorder (like CBT for PTSD) and when the assessment uses standardized measurements (Gastright, 1995; Lilienfeld, 1996/2011; Lohr et al., 1998; Cahill et al., 1999; Coubard, 2012).

Given these methodological aspects, the argument to exhibit scepticism about EMDR treatment can be assessed on five levels.

At level 1, qualitative reports and uncontrolled studies (see “Half the Scientific Community is Enthusiastic About EMDR” Section, level 1) do not rule out effects to be due to factors 1–5.

At level 2, studies comparing EMDR to no treatment (see “Half the Scientific Community is Enthusiastic About EMDR” Section, level 2) neutralize factors 1–2 but not factors 3–5.

At level 3, studies comparing EMDR to non validated treatments (see “Half the Scientific Community is Enthusiastic About EMDR” Section, level 3) control factors 1–4 but not factor 5.

At level 4, one way to conclude about EMDR effectiveness and specificity is to directly compare this treatment to another validated treatment in a Randomized Clinical Trial (RCT). According to Cahill et al. (1999), the first study to achieve this goal was that of Devilly and Spence (1999), that is only 10 years after the original report by Shapiro (1989a,b). The authors showed that CBT-Trauma Treatment Protocol (TTP) was more effective than EMDR in reducing pathology related to PTSD and that this superiority was even more evident by 3-month follow-up (Devilly and Spence, 1999).

Since 1999, further RCTs have shown that EMDR is either as effective as CBT or CBT variants such as CBT-Prolonged Exposure (PE) in adults (Leiner et al., 2012) or CBT-Trauma Focused (TF) in children (Diehle et al., 2015), or less effective than CBT or CBT variants such as CBT-PE in adults (Rothbaum et al., 2005). Other studies have shown that EMDR is more effective than fluoxetine (van der Kolk et al., 2007) and citalopram (Nazari et al., 2011), however pharmacological treatments have not previously been shown to be more effective than CBT treatments in PTSD/trauma. A meta-analysis suggested that EMDR may be more effective than CBT in child PTSD (Rodenburg et al., 2009), but this analysis remains controversial due to weak number of studies (Lilienfeld, 1996/2011).

In other than PTSD/trauma anxiety disorders, EMDR has been demonstrated to be less effective that Participant Modeling (PM; Bandura et al., 1969) in spider phobia using either subjective measures or standardized ones (e.g., Muris et al., 1998).

At level 5, a final way to study EMDR effectiveness is to isolate and examine each of its procedural aspects and their potential effect in a so-called dismantling study (Cahill et al., 1999). As developed in “Procedural aspects” Section, the EMDR protocol involves at least eight components, but the only distinctive feature as compared to other CBT treatments is the therapist-induced bilateral stimulation (Cahill et al., 1999). This issue has been addressed in several studies in PTSD/trauma (e.g., Devilly et al., 1998) and other anxiety disorders such as simple phobia (e.g., Sanderson and Carpenter, 1992), but to date it remains controversial whether eye movements or other laterally alternating stimuli play a role in EMDR effects (Lilienfeld, 1996/2011; Herbert et al., 2000; Davidson and Parker, 2001; Servan-Schreiber et al., 2006; van den Hout et al., 2012).

Taken together, the superiority of EMDR as compared to other validated treatments in PTSD/trauma and other anxiety disorders still remain to be demonstrated. A few studies have evidenced that EMDR is as effective as CBT or CBT variant validated treatments, but there is little evidence that EMDR and its added-value (bilateral stimulation) are more effective than other validated CBT or CBT variants in anxiety disorders.

## Synopsis and Perspectives

### From Psychology

The main reason why the scientific community is divided about EMDR is that its underlying neural mechanism is unknown. As elegantly described by Servan-Schreiber (2003), the current situation of EMDR technique is similar to that of asepsis in Semmelweis’ time: a clinical discovery that lacks a biological demonstration. Austro-Hungarian Ignác Fülöp Semmelweis was a medical doctor working in obstetrics in the 1840’s, who suggested that asepsis during delivery may help to fight germs and decrease the number of mothers’ death due to puerperal fever. The experiments proved that he was right. By cleaning their hands with lime, obstetricians reduced the number of deaths from one third to one twentieth. However medical doctors, judging hand cleaning to be fastidious, got Semmelweis fired from his work, and he was mocked and laughed by his peers until he was confined to a mental institution and dramatically died of ill treatment. Carl Mayrhofer, converted to Semmelweis’ ideas, suggested the microbial hypothesis, which was later confirmed by Pasteur and Lister’s microbiological discoveries. Coming back to EMDR, we may expect the scientific community to be divided on that subject until some scientist draws its neural mechanism on a board, which will make sense for both enthusiasts and sceptics. In the meantime, there are drawbacks to both the enthusiastic and skeptical approaches, which can easily be avoided in future research.

A first issue that is worth mentioning about the enthusiasts is ethical. Many authors are biased in their intentions by the fact that they do business with EMDR, should it be by practicing, supervising, training other practitioners, or selling products for bilateral stimulation. Even when endorsing an academic position and/or not declaring their conflict of interest, they can often be seen using the term “client” instead of “patient”, leaving the reader dubitative about the objectivity of their findings and proposals.

The second type of error by enthusiasts is methodological. In “the Scientific Community is Divided About EMDR” Section, I have reviewed the different aspects of the study designs that need improvement. As previously recommended by Cahill et al. (1999), RCTs should only be conducted comparing EMDR to other validated treatments, which themselves have been previously proved to be effective in PTSD/trauma or other anxiety disorders. Comparing EMDR to either no treatment or to non validated treatments or to validated treatments that are not effective in PTSD/trauma (such as pharmacology) is insufficient. Moreover, such studies should use standardized or quantitative measurements rather than subjective reports like SUDS and VOC.

The third error by the enthusiasts is theoretical. Most explanations to account for EMDR effects (see “Theoretical Hypotheses” Section) are—as is characteristic of certain kinds of psychological theorizing—purely verbal. As such, they do not allow researchers to make predictions about quantitative outcomes that can be linked to underlying physiological mechanisms.

Shortcomings also emerge on the side of sceptics. The first error is historical. Whereas Shapiro made the clinical discovery of EMDR, it is unfair to expect from her that she also unveil its underlying physiological mechanisms. Clinical psychology and basic research in physiology are two different jobs, which are each time consuming and based on specific knowledge and skills, thus usually made by different people. As mentioned above for asepsis and microbes, it usually takes years or decades for a clinical discovery to be elucidated in its intimate mechanisms. Iproniazide initially used as antituberculous drug was found in 1958 by Nathan Kline to have antidepressant properties. But it took years for basic researchers to demonstrate the inhibition of tritiated-noradrenaline uptake by imipramine (Glowinski and Axelrod, 1964). As pointed out by Bernard (1858–1877/1947) “it’s always by chance that everything starts. Science only comes after, and it reasons about what chance has shown” (p. 83)^1^.

The second defect of the skeptics is methodological, by using military veteran stress as a gold standard of PTSD. Contrary to received ideas, I suggest that military posttraumatic stress disorder is not a good model of PTSD, explaining contrasting results between veterans (e.g., Boudewyns et al., 1993; Haagen et al., 2015) and civilians (e.g., Rothbaum, 1997; Rothbaum et al., 2005). Whereas civilian victims most of the time are genuine victims (e.g., rape victims), military soldiers are also active in the process of aggression. By enrolling in the army, they are willing to potentially injure or kill people, who can be enemy soldiers but also civilians in modern wars in which the frontier between the two is vanishing. This particularity renders their psychology more complex and subject to other conditions than anxiety disorders. Additionally, soldiers are more likely to get multi-traumatized than civilians. Taken together, the two populations are presumably very different and should be treated apart.

A final drawback that needs to be mentioned on the skeptical side is to deny that eye movements may have anything to see with emotion, which leads me to the development on the underlying physiological mechanisms of EMDR.

### To Physiology

From the physiological viewpoint, there is no doubt that eye movements can induce emotional shifts, and that reversely emotional shifts can be accompanied by eye movements as motor consequences. Anyone can make this observation and, on that note, writers have as usual preceded scientists. For example, the French writer Guy de Maupassant (1883/2009) published the following: “Back to her bedroom, the young woman [Jeanne Le Perthuis des Vauds] was wondering how two returns to the same places she thought she loved could be so different. Why was she feeling so wounded, why this house, this beloved land, everything that, until now, had made her heart quiver, appeared to be so sad? But her eye suddenly fell onto her clock. The little bee was still fluttering from left to right, and from right to left, with the same fast and continuous movement, above the gilded flowers. Then, abruptly, Jeanne was shaken by a fit of tenderness, moved to tears by this small mechanism that seemed to be so alive, singing the hour to her and beating like a chest.” (de Maupassant, 1883/2009, p. 127)^2^. Even though this is a novel, it is impossible that de Maupassant wrote about the phenomenon of emotional shift associated with eye movements with such acuity without having experienced it himself or observed it in someone else. This observation is not different from that of Shapiro on herself 104 years later.

The reason why eye movements and emotional shifts can be associated as cause or effect is simply that they share common neural circuitry. In a recent article (Coubard, 2015a), I have suggested that eye movements/emotion interaction observed in EMDR may be underlied by both the retino-hypothalamic and retino-collicular (or retino-tectal) visual pathways. In the retino-hypothalamic pathway, a portion of intrinsically photosensitive retinal ganglion cells (ipRGCs) is directed to the supra-chiasmatic nucleus in the hypothalamus (Dhande and Huberman, 2014). This visual system uses melanopsin and projects to the pineal gland, which itself produces melatonin, regulating behavioral and biological functions as well as circadians rhythms related to temperature, wake/sleep, reproduction, autonomic and hormonal functions (Trachtman, 2010). The ipRGCs are also directly linked to the limbic system via their inputs to the amygdala and the habenula (Hattar et al., 2006; LeGates et al., 2012). In the retino-collicular pathway, M1 subtype of ipRGCs targets the superior colliculus (SC), which is itself linked to the amygdala and the orbitofrontal cortex (Krolak-Salmon et al., 2004). Taken together, rhythmic left-right eye movements are able to stimulate the limbic system (i.e., emotional brain) either directly through the retino-hypothalamic system or indirectly through the retino-collicular system. I have also suggested that the way non-visual bilateral stimulation induces emotional shifts may be obtained via supramodal subcortical areas such as the inferior and superior colliculi (i.e., tectal platform; Coubard, 2015a).

To conclude this section, the critical question in EMDR is not to ask whether eye movements and emotion are linked since it is indeed a physiological fact, but rather to determine how a model of attention and bilateral stimulation might explain the complexity of EMDR, and more specifically the way how attention and bilateral stimulation may systematically and effectively enable the processing of trauma-related cognition and emotion.

To address this issue, I suggest that EMDR may gain in being re-examined in integrative models. The Threshold Interval Modulation with Early Release-Rate of rIse Deviation with Early Release (TIMER-RIDER) model, a new framework of motor and cognitive control (see “Towards a Physiological Framework of Motor and Cognitive Control” Section) might be useful to achieve this goal. Before entering the core of this model, I first introduce the reader to the LATER model (Carpenter, 1981, 1999).

## Saccade Reaction Time at a Glance

### Eye Movements

Though eye movements are only one aspect of bilateral stimulation in EMDR, it is worth focusing on their properties given the rich information they provide about brain functioning. To explore the visual world, humans make a variety of eye movements. Version or conjugate eye movements refer to movements of the two eyes in the same direction (to the left or to the right, up or down, or in any oblique direction). In vergence or disconjugate eye movements, the eyes move in the opposite direction: they converge at close or diverge at far. Both version and vergence eye movements can be either step (they are saccades in direction and step vergence in depth, respectively) to jump from one area of the visual scene to another one, or smooth (pursuit and vergence) to follow a target moving in direction or in depth. Besides intentional movements, the eyes also move in a reflex way to stabilize images on the retina during head movements (vestibulo-ocular reflex) or to track a moving visual pattern (optokinetic reflex). Even when the eyes fixate a single point and seem stationary, they are never at rest and continuously move through micromovements—tremor, drifts and microsaccades—which are crucial for vision (Coubard, 2011, 2015b).

Every (little or large) movement offers researchers a variety of behavioral parameters for their research purpose: reaction time or latency, duration, amplitude, gain, peak velocity.

Saccades are extraordinary movements as their stereotyped behavior provides reliable measures subjected to detailed and quantitative analysis, while their underlying neural circuitry forms a well-known system that offers insight into brain functioning (Carpenter, 2004). In this section, let’s keep an eye on saccade reaction time ( SRT), the time elapsed between target onset and saccade initiation.

### Reaction Times on a Traditional Frequency Histogram

**Figure 1:**
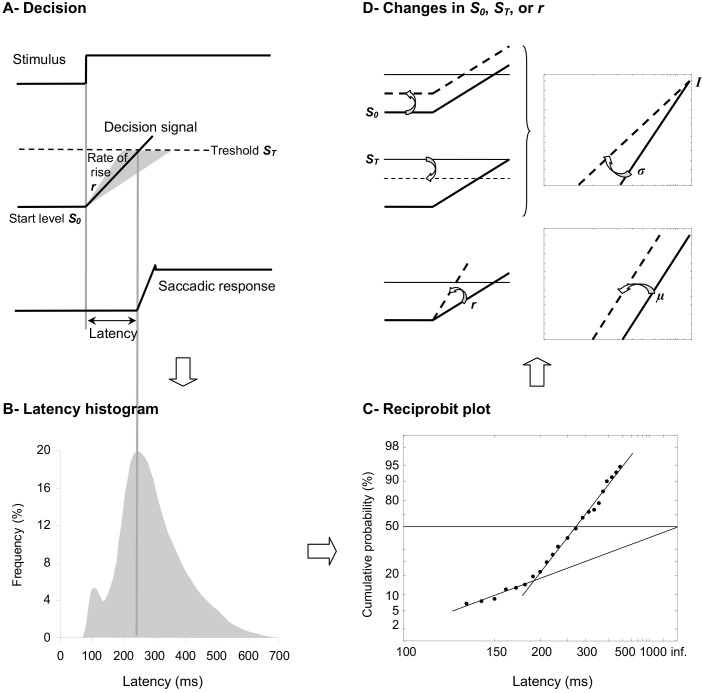
**(A) LATER model.** From bottom to the top, we show the saccade position signal, the theoretical decision signal in a LATER unit, and the stimulus signal. In response to the Stimulus, the Decision signal initiates at the Start level S_0_, increases at a Rate of rise *r*, until it reaches the Threshold S_T_ at which the Saccadic response begins. **(B)** Traditional histogram of Frequency (in %) as a function of Latency (in ms). The main distribution is skewed with a tail to the right and some population of express saccades can exhibit a distinct peak. **(C)** Resulting reciprobit plot of Cumulative probability on a probit scale (in %) as a function of Latency on a reciprocal scale (in ms). The main recinormal distribution lies on a straight line described by μ and σ. Early saccades can occur lying on a second line described by σ’. **(D)** Changes that can occur in LATER. A change in the distance between S_0_ and S_T_ results in a swivel of the recinormal distribution around the intercept (up). A change in the rate of rise *r* results in a lateral shift of the recinormal distribution (down).

On a traditional frequency histogram (see Figure 1B), SRT has been classified into different clusters supposed to represent distinct subpopulations of saccades. In healthy young adults, express, fast regular, slow regular, and late latencies correspond to SRT of 80–134 ms, 135–179 ms, 180–399 ms, and over 400 ms, respectively (Fischer et al., 1997). Eliciting one or the other subpopulation of saccades can be done by manipulating timing conditions between fixation point offset and target onset. As example, introducing a temporal gap between the two events favors the appearance of express saccades first observed in 1983 in monkeys (Fischer and Boch, 1983) and a year later in humans (Fischer and Ramsperger, 1984). Whether such phenomenom is due to either preparation or the release of attention-inhibition is still under debate (Paré and Munoz, 1996; Findlay and Walker, 1999; Isa and Kobayashi, 2004). Observing the proportion of saccades in the different clusters of SRT can be a tool to assess the effects of any intervention. Indeed, I demonstrated that a training of only 2.5 months in fall prevention rehabilitated the express triggering (80–134 ms) of elderly fallers (Coubard, 2012), which was previously shown to be rare in this population (Yang et al., 2008). However the use of traditional frequency histograms for examining SRT has some limitations. As emphasized by Carpenter (1981, 1999), one of them is that the overall shape of SRT distribution is often asymmetric with a longer tail to the right, which does not fit standard mathematical models. This led the author to the point that researchers may not, through SRT, measure the right thing (Carpenter, 1981, 1999).

### Reaction Times on a Reciprobit Plot

Taken together with the fact that SRT is surprisingly long and variable from trial to trial, Carpenter (1981, 1999) suggested that SRT may reflect, rather than the time needed for neural circuitry to transfer the information, a cascade of decisional mechanisms resulting in movement procrastination. In this context, dynamic Bayesian models may be more likely to put forth our knowledge of movement. In his LATER model standing for *Linear Approach to Threshold with Ergodic Rate* and reminding the procrastinative effect, Carpenter (1981, 1999) suggested that before the movement is performed in response to a visual stimulus, some kind of decision signal starts at a level S_0_ (the initial threshold), before it rises at a constant rate *r*, until it reaches a level S_T_ (the final threshold), at which point the decision is made and the movement is initiated (see Figure 1A). Since reaction time and rate are reciprocally related, Carpenter (1981, 1999) suggested to measure not the distribution of SRT (see Figure 1B), but that of its reciprocal 1/SRT, which is called *promptness* (see Figure 1C). As soon as the distribution of promptness is Gaussian and using two special scales for its graphic representation, a reciprocal scale in the *x* axis and a probit scale in the *y* axis, the so-called recinormal distribution results in a straight line, which can be parsimoniously described by only two parameters, its median μ and its slope σ (see Figure 1C). Occasionally, early SRTs may occur, which lie on a second line that is described by only one parameter, its slope σ’ (Carpenter, 1981, 1999; see Figure 1C).

More interestingly for our purpose, two types of changes can occur in LATER model, which provides researchers direct information about underlying central motor control or decisional mechanisms (Carpenter and Williams, 1995; Reddi and Carpenter, 2000; see Figure 1D). The recinormal distribution can either swivel around its intercept (i.e., the origin value in the *y* axis, which is rightward as a consequence of the reciprocal scale in the *x* axis; see Figure 1D, Up), or shift laterally without any change in its slope (see Figure 1D, Down). Leftward or rightward swivelling/shift indicates some decrease or increase in SRT, respectively. Importantly, a swivelling indicates that a change has occurred in the distance between S_0_ and S_T_, while a shift indicates that a change has occurred in the rate of rise of the decisional signal—also called the gain. Specifically, a leftward or rightward swivelling indicates that the distance between decisional thresholds has decreased or increased, respectively. A leftward or rightward shift indicates that the gain has increased or decreased, respectively (Carpenter and Williams, 1995; Reddi and Carpenter, 2000; see Figures 1A–D).

Researchers have here a tool to test any type of intervention, pharmacological (Michell et al., 2006), surgical (Temel et al., 2008), by training (Coubard, 2012), and offer insight into how such intervention has acted onto motor control and its decisional mechanisms. Such model can be used not only for SRT but also for reaction times of any human movement, should it be of the eye, of the head, of the arm, of the leg, etc. This opens the perspective of exciting future research in the field of eye and body movements.

## Towards a Physiological Framework of Motor and Cognitive Control

### Bridging Attention to Decision

I recently introduced a new framework to bridge attention and decision models, which may be useful to re-examine EMDR underlying mechanisms—TIMER-RIDER model (Coubard, 2012). This model is based on a model of saccade and vergence initiation (Coubard, 2011) and the LATER model (Carpenter, 1999). It is now consensual that attention involves three main components: vigilance, selection, and control. In this model, our motivation to focus on attentional control is twofold. First, control is currently the less known component of attention. Second, it is thanks to control and its underlying prefrontal network that humans voluntarily drive and act onto the impetuous horse that represents their whole attention system. In cognitive sciences, attentional control is defined as the ability to maintain goal-directedness over time in the face of distraction, temporarily stopping the activity to respond to other information, and coordinating the course of concurrent activities (Parasuraman, 2000).

Theoretically, attentional control has been described in temporal or top-down theories as a supervisory system gathering multiple cognitive processes (e.g., Norman and Shallice, 1986; Fuster, 1989; Stuss et al., 1995). The neural basis of attentional control within the prefrontal cortex has been identified for at least three of these processes, namely energizing, setting and monitoring (Stuss, 2011), whereas for other processes (e.g., inhibiting), it seems to be more distributed in the brain (Munakata et al., 2011). In the meanwhile, theories of decision have described a random walk process determined by the distance to the thresholds and the accumulation of evidence (e.g., Ratcliff, 1978; Carpenter, 1981; Schall, 1995).

**Figure 2:**
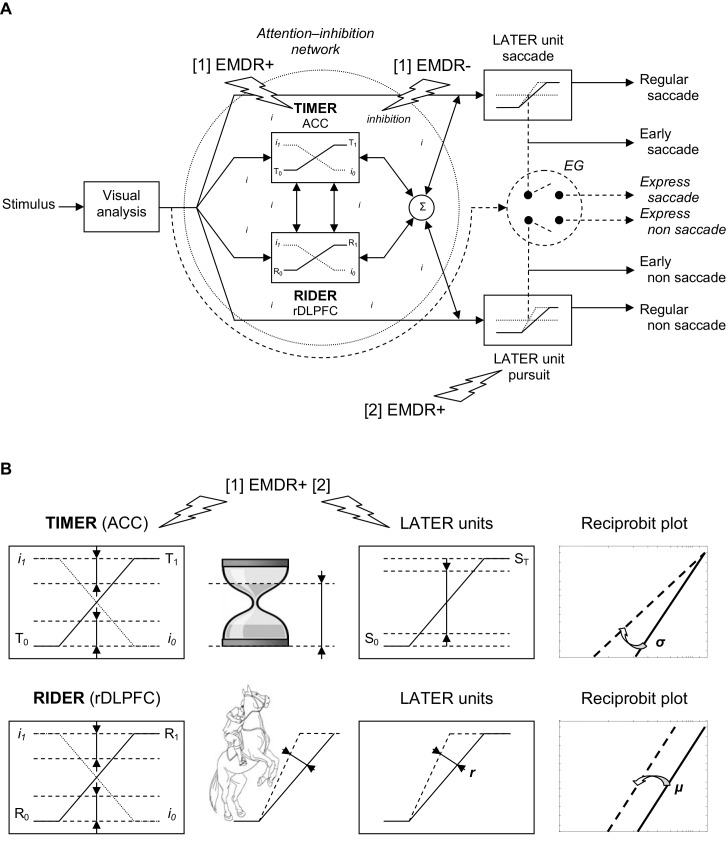
**(A) TIMER/RIDER model.** Visual information from the two retinas is analyzed to determine the spatial location of the stimulus. **Excitatory mechanisms** (right). Once the decision is made to elicit an eye movement toward the stimulus, decision signals initiate in LATER units for saccade and non saccade, which rise at a constant rate to reach the threshold at which regular or early saccade and/or non saccade are triggered. In LATER units, the thresholds and/or the rates of rise for saccade and non saccade can differ. **Inhibitory mechanisms** (dotted circle). In a global attention-inhibition network, the inhibition process has value between I_1_ and I_0_ throughout the network. **Modulation**. Two units modulate excitatory and inhibitory mechanisms: TIMER (Threshold Interval Modulation with Early Release) corresponding to anterior cingulate cortex (ACC) activity, and RIDER (Rate of rIse Deviation with Early Release) corresponding to right dorsolateral prefrontal cortex (rDLPFC) activity. TIMER and RIDER signals increase from respectively values T_0_ and R_0_ to values T_1_ and R_1_ (full line), causing a mirror decrease in inhibition from I_1_ to I_0_ (dotted line). Modulators’ effects are added (σ) producing a change in LATER units in either decision thresholds or in decision gain or in both. In turn, movement-induced LATER activity can stimulate TIMER and RIDER activity and reduce the level of inhibition. **Early vs. express triggering** (right). Under strong conditions of attention-inhibition release by TIMER/RIDER modulators, LATER units produce short-latency eye movements in the form of early movements. Under optimal conditions of attention-inhibition release, the short visual route bypassing attentional/decisional mechanisms (dashed arrow) elicit express movements in an all or nothing way (switch). **(B) Effects of TIMER/RIDER on the inhibition process and on LATER units**. An increase in the TIMER signal (T) causes a mirror reduction in the inhibition process I, and a reduction in the distance between initial threshold S_0_ and final threshold S_T_ of LATER units, resulting in a swivel of the recinormal distribution. TIMER also recalls an hourglass with decreasing distance between the level of sand in its upper part and the baseline of its support. An increase in the RIDER signal (R) also causes a mirror reduction in the inhibition process I, and an increase in the rate of rise *r* in LATER units, resulting in a shift of the recinormal distribution. RIDER is reminiscent of a horseman spiriting his mount to enhance the slope of the rearing up. **(A,B)** **Eye Movement Desensitization and Reprocessing (EMDR) effects**. EMDR may have two actions in parallel: [1] EMDR may boost (+) TIMER modulator and inhibit (−) the inhibition process, resulting in higher decision-making and shortened reaction times; [2] EMDR may boost (+) LATER unit for pursuit and retroactively stimulate TIMER/RIDER activity and reduce the level of inhibition, with a similar result on decision and reaction times.

TIMER-RIDER is a framework that aims at bridging attention and decision by emphasizing two processes, inhibition and decision, and introducing two modulators for regulating these processes, so-called TIMER and RIDER (Coubard, 2012). The model is illustrated in Figure 2.

### Excitatory Modules and Inhibitory Network

In this model, excitatory mechanisms for movement activation are embodied in LATER units. Considering the visuomotor modality, visual information from the retina of both eyes is processed to build a percept of stimulus location in space. As soon as the visual stimulus jumps from one location to another one, the LATER unit devoted to saccade will be activated (see Figure 2A, Right/up). As previously described, the decision signal for saccade starts at an initial threshold S_0_, rises at a constant rate *r*, until it reaches a final threshold S_T_, at which point a saccade is initiated (Carpenter, 1999). There exist other LATER units for other types of movement: non saccade eye movements such as pursuit, vergence, etc. Each LATER unit for a specific movement has specific thresholds and gain (Takagi et al., 1995; see Figure 2A, Right).

Inhibitory mechanisms for movement suppression take the form of a global network lodging two modulators to regulate both the inhibition process and the LATER units (see Figure 2A, Left). Attention-inhibition is hypothesized to be more global consistent with neurophysiological findings showing that there is not a specific inhibitory module for each LATER unit (Coubard, 2012). In this network, the inhibition process can take any value between I_1_ corresponding to maximal inhibition for which the movement is completely suppressed, and I_0_ corresponding to minimal inhibition for which the movement is completely released. In this way, the attention-inhibition network does not trigger movement on its own but rather modulates the triggering of movement in time, by shortening or enhancing its reaction time.

### TIMER and RIDER Modulation

To regulate movement, the attention-inhibition lodges two modulators: TIMER and RIDER. TIMER stands for *Threshold Interval Modulation with Early Release*. It allows LATER units to reduce the distance between the initial and final thresholds S_0_ and S_T_ of the decision signal, which results in movement with shortened reaction time (see Figure 2A, Left and Figure 2B, Up). Importantly, TIMER acts onto all LATER units, thus onto all types of movement. On the other hand, RIDER, standing for *Rate of rIse Deviation with Early Release*, enhances the rate of rise of the decision signal in LATER units, which results once again in movement with earlier latency. Similarly, RIDER acts onto all LATER units, therefore on all movements (see Figure 2A, Left and Figure 2B, Down). The two modulators can interact and their effects are combined to produce in LATER units some modulation of either the distance between thresholds or the gain or both. On their own, they cannot activate or suppress movement, but only modulate their triggering (Coubard, 2012).

Importantly, the rate of rise of TIMER (T) and RIDER (R) signals is supposed to be linearly and inversely correlated to the Inhibition Process (I). In other words, the increasing activity of TIMER and of RIDER causes a direct decreasing activity of the inhibition process. Reversely, a higher inhibition process causes the reduction of TIMER and of RIDER activities (Coubard, 2012; see Figure 2A, Left and Figure 2B).

Thus, TIMER and RIDER have the ability to modulate the subject attentional state, leading to attentional fluctuations over time depending on their level of activity. We suggest that such attentional state may be influenced by internal or external contingencies, such as emotional disorder or therapeutic intervention. The TIMER-RIDER model also formalizes some distinction between express vs. early movements (see Figure 2A, Right). Both express and early movements have short reaction times, but they may correspond to distinct underlying mechanisms. Indeed, express movements correspond to movements that completely bypass the attention-inhibition network. In humans, such movements have been described for ocular saccades (Fischer and Ramsperger, 1984) and for vergence (Coubard and Kapoula, 2006). They are observed under specific psychophysical conditions (for a review, see Findlay and Walker, 1999), or naturally in developmental (Cavegn and Biscaldi, 1996) or psychiatric (Currie et al., 1993) disorders. In contrast, early movements might result from a high state of attention-inhibition release allowing for short reaction time movements that are nevertheless still under attentional control (see Figure 2A, Right). I have suggested that it would be the function of TIMER and RIDER to make possible such high level of attention-inhibition release, by reducing the distance between thresholds and the gain of the decision signal (Coubard, 2012).

In summary, I propose that TIMER-RIDER model may be seen as physiological framework of motor and attentional control. Indeed, Claude Bernard was already writing from 1866 that “the brain exerts on reflexive movements a certain influence that we have characterized already, by calling it moderative action. But there is also a directive action.”^3^ (Bernard, 1866, pp. 369–370). In TIMER-RIDER terms, Bernard’s moderative action may correspond to the inhibition process, while Bernard’s directive action may fit the decision process.

How such a model reflects physiological reality in the human brain and how it may contribute to our understanding of anxiety disorders and of EMDR mechanism are the issues we now discuss.

## An Integrative Model for the Neural Mechanism of EMDR

### Physiological Implementation of Excitatory Modules and Inhibitory Network

The eyes move thanks to oculomotor muscles, which are innervated by motor neurons, which are themselves innervated by burst neurons in the brainstem. Different burst neurons control the subtypes of eye movements. As example, burst neurons for horizontal saccades are located in the paramedian pontine reticular formation (PPRF; Fuchs et al., 1985), while those for vertical saccades lie in the rostral interstitial nucleus of medial longitudinal fasciculus (riMLF; Büttner-Ennever and Büttner, 1978; King and Fuchs, 1979). Burst neurons generate three forces: the pulse is the velocity command to rotate the eye; the step is the position command to maintain the eye in its new position; the slide is embedded between the two to counteract viscoelastic forces of the oculomotor plant (for reviews, see Scudder et al., 2002; Coubard, 2013). In terms of LATER model, motor neurons and premotor neurons may not participate to the decision signal (Boucher et al., 2007). In contrast, long-lead burst neurons (LBBN) in the brainstem (Kaneko, 2006), movement neurons in the SC (Dorris and Munoz, 1998), in the caudate nucleus (Lauwereyns et al., 2002), and in the frontal eye field (FEF; Hanes and Schall, 1996) show a progressive increase in their firing rate prior to the activity of burst neurons, consistent with LATER model. Therefore, we suggest that the different LATER units are embodied by LBBNs and movement neurons from the SC to the FEF. As such, LATER units represent excitatory mechanisms for selective attention and movement activation in TIMER-RIDER model.

For inhibitory mechanisms, the attention-inhibition network involves different areas from the brainstem to the prefrontal cortex. Premotor neurons are under the inhibitory control of omnipause neurons (OPN) located in the pontine raphe (Scudder et al., 2002; Coubard, 2013). OPNs commonly inhibit burst neurons for horizontal and vertical saccades (Scudder et al., 2002), as well as for vergence (Mays et al., 1986). But OPNs may not participate to the attention-inhibition network as they are modulated during the ballistic period of movement (Boucher et al., 2007). Rather, TIMER-RIDER attention-inhibition network may be embodied by fixation neurons that have been found in the SC (Munoz and Wurtz, 1993), the substancia nigra (Hikosaka and Wurtz, 1983), the FEF (Segraves and Goldberg, 1987), and the dorsolateral prefrontal cortex (DLPFC; Tinsley and Everling, 2002). The inhibition process (the I signal) would fit the activity of fixation neurons, which decrease their firing rate prior to saccade initiation. The reason why the attention-inhibition network is supposed to be more distributed than LATER units is that fixation neurons do not systematically respond to local LATER units, which can have common or several sources of inhibition. As example, SC movement neurons are inhibited by fixation neurons of SC (Munoz and Wurtz, 1993), of substancia nigra (Hikosaka and Wurtz, 1983) and of DLPFC (Goldman and Nauta, 1976; Leichnetz et al., 1981; Gaymard et al., 2003).

### Physiological Implementation of TIMER and RIDER Modulation

How TIMER and RIDER modulation of LATER units may be implemented in the brain has been revealed by an fMRI study examining the neural correlate of LATER model (Domenech and Dreher, 2010). The authors showed that the anterior cingulate cortex (ACC) activity was positively correlated to the distance to the decisional threshold, but not to the slope of the accumulation of sensory evidence, whereas the right DLPFC (rDLPFC) showed the reverse pattern, i.e., was positively correlated to the decision gain but not to the distance to the decisional threshold (Domenech and Dreher, 2010).

In terms of TIMER-RIDER model, this suggests that the TIMER signal (T) for modulating distance to decision threshold approximates ACC activity, while the RIDER signal (R) for modulating decision gain fits rDLPFC activity (see Figure 2A, Left and Figure 2B).

### Application to Emotional Disorders

Let’s now examine how TIMER-RIDER framework helps to understand further PTSD and its treatment by EMDR.

It is well established that emotion and attention are tightly linked such that acting on one influences the other (for a review, see Brosch et al., 2013). Anxiety disorders alternate between two main tendencies of imbalance between emotion and attention: emotional bypass and cognitive stifling (Servan-Schreiber, 2003).

Emotional bypass occurs whenever emotion overcomes attention, and takes different forms from mindless emoting and hypocontrol (Greenberg and Safran, 1987; Shapiro, 2001) to panic disorder and PTSD (Servan-Schreiber, 2003). In monkeys, Arnsten and Goldman-Rakic (1998) showed that stress (a loud noise) can take prefrontal cortex cognitive function off-line through a hyperdopaminergic mechanism. In terms of TIMER-RIDER model, such deficit is equivalent to bypassing the entire attention-inhibition network leading to express reaction times (see Figure 2). Indeed, parallel to the long route of decisional mechanisms, the retino-collicular pathway acts as a short route that enables SRT from 60 ms in monkeys (Fischer and Boch, 1983) and 80 ms in humans (Fischer and Ramsperger, 1984). The SC is critical for express triggering (Schiller and Tehovnik, 2005), where a switch enables visual neurons to directly activate motor neurons (Isa and Kobayashi, 2004). Taken together, these studies explain how express eye movements can be biomarkers of control loss observed in psychiatric disorders such as schizophrenia (Currie et al., 1993) or bipolar disorder (Velasques et al., 2013).

Cognitive stifling occurs whenever attention overcomes emotion, and takes various forms from feeless thinking and hypercontrol (Greenberg and Safran, 1987; Shapiro, 2001) to depression (Servan-Schreiber, 2003). Such emotional deficit is associated with decision-making difficulties (Brosch et al., 2013). In terms of TIMER-RIDER model, such deficit is equivalent to an abnormally high level of inhibition process (I signal) within the attention-inhibition network, resulting in low levels of TIMER and/or RIDER activities and long reaction times (see Figure 2).

### Application to EMDR

In this context, the goal of EMDR in PTSD is to restore some balance between these two tendencies, over-emotion and over-attention, to lead to the state of harmonious flow of cognition, emotion and physical sensations (Servan-Schreiber, 2003). Here, we suggest that EMDR, thanks to its multiple procedure components, achieves this goal in different ways, which can act in parallel.

My first suggestion is that EMDR may boost the TIMER modulator and ACC activity (see Figures 2A,B, [1] EMDR+). This effect may be obtained by asking the patient to focus his/her attention on the different aspects of the PTSD, should it be the visual image, the emotion associated with the image, and the physical sensations associated with the emotion. In this way, the therapist stimulates one aspect of attentional control, energizing, which is a general increase in attentional control level (Stuss et al., 2005). Lesion and brain imaging studies have shown that energizing is correlated with the activity of dorsomedial prefrontal cortex (Stuss, 2011). With respect to TIMER-RIDER, enhancement of energizing or TIMER activity leads to a mirror decrease in the inhibition process in the attention-inhibition network (see Figure 2A, [1] EMDR−). This also results in higher distractibility, which is consistent with distraction hypothesis about EMDR effects. In LATER units, the effect of higher TIMER activity and of reduced inhibition is to reduce the distance between initial and final decision thresholds, leading to higher decision-making skill and shortened reaction times.

Functional brain imaging studies are compatible with this suggestion. Indeed, a meta-analysis of neuroimaging of emotional processing in PTSD and other anxiety disorders has evidenced hypoactivation in the dorsal and rostral ACC and ventromedial prefrontal cortex-structures linked to the regulation of emotion (Etkin and Wager, 2007). Additionally, a SPECT study showed that EMDR intervention in six PTSD patients led to hyperactivation of ACC and left frontal lobe (Levin et al., 1999). The activation of left frontal lobe, which is associated with setting attention (Stuss, 2011), might be the result of EMDR-induced changes in problem solving ability.

Whether EMDR may also boost the RIDER modulator and rDLPFC activity is more hypothetical. As previously mentioned, bilateral stimulation may help to modify to generate imbalance in automatisms (Shapiro, 1991; MacCulloch and Feldman, 1996). It is the function of rDLPFC to monitor the level of activity of schemata, while the attentional process that enables automatic processes to work more smoothly—so-called adjusting contention-scheduling—has hitherto not been associated to any specific area within the prefrontal cortex (Stuss et al., 2005; Stuss, 2011). Additionally, brain imaging studies of anxiety disorders or of EMDR effects have not shown any specific activation of rDLPFC. The way that automatisms are imbalanced by EMDR may not be obtained by monitoring or adjusting contention-scheduling. Rather, such imbalance in automatisms may be a consequence of higher TIMER activity and higher distractibility in the attention-inhibition network.

An easy way to test my prediction will be to measure SRT before and after an EMDR procedure. If my suggestion is correct, EMDR by boosting TIMER rather than RIDER should result in a swiveling rather than a shift of SRT recinormal distribution (see Figure 2).

Finally, TIMER-RIDER is also compatible with an effect of bilateral stimulation on its own, particularly of overt or covert eye movements *per se*. Indeed in this framework, any overt movement of the eyes that is induced by the therapist should be able to boost LATER units, particularly LATER units for pursuit in EMDR visuomotor modality (see Figures 2A,B, [2] EMDR+). Additionally, the instruction of following a moving target with accuracy and speed is sufficient to improve reaction times. In non-visuomotor modalities (e.g., auditory, tactile), there might be some subliminal stimulation of LATER units for bilateral movement and future investigation is needed to objectively track either overt movements or covert micromovements in the absence of visuomotor stimulation. In turn, either overt or covert movement stimulation reduces distances between decision thresholds and enhances decision gains of LATER units. As a result, movement stimulation on its own retroactively acts onto the attention-inhibition network by reducing the inhibition process and by boosting TIMER and RIDER activities. Thus, our final suggestion is that bilateral stimulation on its own, either overt or covert, also participates in EMDR underlying mechanism.

## Conclusion

The different aspects of EMDR procedure as well as hypotheses and theories accounting for its beneficial effects have put emphasis on attentional control in the process of targeting the emotional deficit. Indeed, EMDR seems to restore some balance in attention and emotion between two tendencies of anxiety disorders, over-emotion vs. over-attention. In the framework of movement models such as the TIMER-RIDER—model, EMDR might restore some balance between two types of responses, uncontrolled vs. over-controlled movements. Such effect may be obtained by two processes acting in parallel: activity level enhancement of a specific component of attention control, presumably energizing or TIMER modulator; and bilateral stimulation in any sensorimotor modality. The two processes result in lower inhibition and higher distractibility enabling dysfunctional information to be integrated. The TIMER-RIDER model, by integrating theories of EMDR, eye movement neurophysiological findings, and functional brain imaging of PTSD and of EMDR intervention, may be a useful integrative model to study attentional and/or emotional disorders, such as anxiety disorders.

## Author Contributions

OAC confirms being the sole contributor of this work and approved it for publication.

## Conflict of Interest Statement

The author declares that the research was conducted in the absence of any commercial or financial relationships that could be construed as a potential conflict of interest.

## Footnotes

^1^The original text in French is “C’est toujours par hasard que tout commence. La science ne vient qu’après et elle raisonne sur ce que le hasard a montré.”

^2^The original text in French is the epitaph.

^3^The original text in French is “[L]e cerveau exerce sur les mouvements réflexes une certaine influence que nous avons caractérisée déjà, en l’appelant action modératrice. Mais il y a aussi une action directrice.”
